# Evaluation of the effectiveness of health education on first aid with the provision of first aid kits among rural households of India: A quasi-experimental study

**DOI:** 10.1177/22799036261427950

**Published:** 2026-03-23

**Authors:** Amit Kumar Rao, Rahul Hegde, Nanjesh Kumar Siddappa, Abiga George, Sanjay Kini Bailur, Harshitha Hanglur Narasimha

**Affiliations:** 1 Nitte (Deemed to be University), KS Hegde Medical Academy, Mangalore, Karnataka, India; 2 Department of Public Health, Karnataka State Rural Development and Panchayat Raj University, Gadag, Karnataka; 3 Department of Community Medicine, Kasturba Medical College, Manipal Academy of Higher Education, Manipal, Karnataka, India

**Keywords:** first aid, health education, wounds and injuries, emergencies, rural health

## Abstract

**Introduction:**

The World Health Organization indicates that injuries contribute to an estimated 4.4 million deaths each year, representing nearly 8% of all deaths globally. These figures underscore the critical need for preventive measures, especially considering the wide range of injury types and their severe consequences. First aid education is one of the most effective methods for preventing injury-related morbidity and mortality in the community. Yet, despite its importance, there remains a significant gap in its implementation.

**Methods:**

A community-based, uncontrolled before-after intervention study was conducted among 400 participants. After baseline assessment of first aid knowledge and skills, participants received a combined intervention consisting of a 1-h first aid training session and provision of a first aid kit. After 3 months, their knowledge and skills were reassessed to determine the effectiveness of the intervention.

**Results:**

In the 80-point knowledge assessment, the study showed a statistically significant improvement in participants scores (*p* value < 0.001). There was a statistically significant improvement across age, sex, educational level, family income, and occupation. In the 100-point skill assessment tool, the median score improved from 39 (IQR 37.8, 48.9) before the intervention to 87 (IQR 84, 88.9) after the intervention (*p* value = 0.018).

**Conclusion:**

First aid health education, combined with the availability of first aid kits, effectively increased participants’ knowledge and skills in managing emergencies. These findings highlight the importance of targeted interventions to enhance emergency preparedness across diverse populations in low-resource settings.

## Significance for public health

Injuries remain a leading cause of morbidity and mortality worldwide and disproportionately affect populations in low- and middle-income countries. Rural households, in particular, face barriers to timely medical care, making immediate first aid a crucial determinant of outcomes in emergencies. This study shows that structured health education, combined with the provision of first aid kits, substantially improves knowledge and practical skills among rural community members. By equipping households with both the competence and resources needed to respond to injuries, such interventions can bridge critical gaps in pre-hospital care and reduce complications from common emergencies such as cuts, burns, falls, and snake bites. The intervention’s effectiveness was consistent across age, sex, education, and income groups, highlighting its applicability in diverse populations. Scaling up such initiatives through primary health care systems and community health workers could strengthen community resilience, enhance emergency preparedness, and ultimately reduce preventable injury-related deaths and disabilities.

## Strengths of the study

- While most studies focus on providing first aid training to students, teachers, and health workers, the present study focused on household members in rural areas who commonly encounter injuries and illnesses in their households and neighborhoods.
- Very few studies have assessed first aid training combined with provision of first aid kits to each household to address the logistical aspects of delivering first aid.
- First aid health education, combined with the availability of first aid kits, effectively increased participants’ knowledge and skills in managing emergencies, underscoring the importance of targeted interventions to enhance emergency preparedness across diverse populations.

## Introduction

The World Health Organization (WHO) estimates that intentional and unintentional injuries cause about 4.4 million deaths each year, representing about 8% of all deaths globally.^1^ Road traffic accidents, falls, drowning, poisoning, burns, fractures, nosebleeds, snakebites, and violence are among the most common types of injuries.^1^ In India, lacerations have been reported as the most common household injuries (57.1%),^2^ and in Nepal, falls (48%) followed by burns (17.4%) are the two most commonly occurring injuries.^3^ Other studies have found falls, burns, and cuts to be the most common injuries in households in India, Saudi Arabia, and Kuwait.^4–6^ These data point to the urgent need for effective injury prevention strategies, particularly in household settings, where the majority of such injuries occur.

Training the public in first aid techniques can be an effective way to reduce injury-related disability and deaths.^7^ First aid is defined as the initial, immediate assistance provided to individuals with minor or serious injuries or illnesses until professional help arrives.^8^ Administration of first aid involves simple techniques that require minimal or no equipment but can be lifesaving. Despite its importance, significant gaps in knowledge and skills in first aid persist in the population. One study from Saudi Arabia found that only 36% of people had received first aid training.^9^ Several studies have reported that first aid knowledge and training are inadequate in countries such as India, Ethiopia, Jordan, and Saudi Arabia.^5,10–15^ Insufficient understanding of first aid procedures can lead to adverse health outcomes and complications, which disproportionately affect vulnerable groups such as children and older adults, who are more commonly affected by burns, falls, and road traffic accidents.^16^

In rural India, where access to emergency medical services is limited, training in first aid can be even more vital.^17^ A well-stocked first aid kit in rural households can address common agricultural injuries such as cuts, burns, and fractures resulting from limited safety measures. However, only a minority of homes keep first aid kits; for example, one study from China reported that only about 25% of households had a kit available.^18^

This study aimed to enhance first aid knowledge and skills among rural households through health education delivered by trained personnel using first aid kits and audiovisual tools. Unlike most programs targeting first responders, teachers, or students, this intervention focused on rural household members who frequently face injuries and illnesses at home or in their communities. By providing each household with a first aid kit alongside education, the study also addressed practical barriers to first aid delivery. Its effectiveness was evaluated after 3 months by assessing changes in first aid knowledge and the actual use of the kits.

## Methodology

**Study design:** A community-based, quasi-experimental before-after intervention study.

**Study duration:** April 2023 to March 2025.

A survey was conducted among 50 rural households in the district approximately 3 months before the start of the pilot study to determine the most common injuries and incidents in the study population. The most common incidents were finger injuries from kitchen knives, injuries due to falls, injuries due to slipping on the floor, steps, burns, and snakebites. The training topics were selected based on this survey and included detection and initial management of cuts, bruises, falls, fractures, epistaxis, burns, dehydration, seizures, hypoglycemia, snakebites, choking, and foreign bodies in the eye.

**Study tools:** To provide first aid health education on the above topics, audiovisual aids from St John Ambulance’s first aid demonstration videos and the St John Ambulance’s manual on First Aid, 11th edition, were used to prepare the study tools.^19^ These included a knowledge assessment questionnaire, a skill assessment checklist, audiovisual aids, and charts for training, along with a first aid kit for demonstration at individual households. A questionnaire was developed for pre- and post-intervention assessment of first aid knowledge, scored out of 80 points. For example, according to the manual, first aid for epistaxis consists of four steps: reassuring the person, asking the person to pinch the nose with index finger and thumb, leaning forward, and breathing through the mouth. Each of these four steps was allocated one point, giving a total of four points for epistaxis management. Similarly, all other topics (cuts, bruises, falls, fractures, burns, dehydration, seizures, hypoglycemia, snakebites, choking, and foreign bodies in the eye) were scored based on the number and importance of steps in their management. Skill assessment was done using scenarios and simulations, scored with a 100-point checklist for topics such as management of abrasion, cut injury, burns, foreign bodies in the eye, ORS preparation, laceration, and fall from height. Subject experts validated the questionnaire and checklists for content and feasibility. The study tools were pilot tested on 20 households (Cronbach’s alpha = 0.72), and relevant changes were made. Inter-rater reliability for pre- and post-intervention knowledge scores was assessed using a random-effects model with absolute agreement. The intraclass correlation coefficient (ICC) was 0.805 (95% CI 0.66–0.9, *p* < 0.001) for pre-intervention and 0.921 (95% CI 0.86–0.98, *p* < 0.001) for post-intervention, indicating good to excellent agreement between raters. Identical first aid kits (Appendix 1) were provided to each household after health education tailored to the management of the 12 conditions.

**Sample size:** A pilot study conducted on 20 participants yielded an effect size of 0.15. Using this as a reference, with a 5% level of significance, 80% power, and a paired t-test formula, the required sample size was estimated to be 351. Allowing for a 10% loss to follow-up, the final sample size was set at 400. Although training was provided to all household members present at the time of the visit, pre- and post-tests were conducted on a single member in each household. This member was selected randomly by writing names on slips of paper and drawing one by lottery.

**Study population:** The study was conducted in the coastal districts of Dakshina Kannada and Udupi in Karnataka state. Ten villages were selected from each district by simple random sampling (SRS), and within each village, 20 houses were again selected using SRS. Only one participant was enrolled from each household to increase population representation within villages.

**Inclusion criteria:** The study included adults older than 18 years who were present in the household at the time of the study visit.

**Exclusion criteria:** Households with adults working in the health sector or members with prior education in first aid were excluded.

**Ethics review:** Ethical clearance was obtained from the Institutional Ethics Committee of Nitte (Deemed to be) University.

**Data collection:** Nurses and medico-social workers who delivered the first aid training were trained by certified doctors experienced in first aid. Written informed consent was obtained from participants after explaining the study’s purpose and procedures. A participant information sheet was provided to each household with basic information about the research and the contact numbers of the study investigators. After collection of sociodemographic data and baseline knowledge and skills assessment by the investigators, first aid health education was provided to household members by a team consisting of a nurse (Bachelor of Science in Nursing) and medico-social workers. Each session lasted 60 min, including 45 min of interactive training using audiovisual aids, charts, and demonstrations, followed by 15 min for questions and clarifications. After the training session, each household received a first aid kit. Three months after the intervention, the investigators reassessed the first aid knowledge and skills in managing common injuries and illnesses using questionnaires, checklists, and simulations.^20^ During these 3 months, no additional interventions were provided by the study team, and there were no external activities such as health campaigns or training that could influence the outcomes.

**Data analysis:** Data were compiled using Microsoft Excel and analyzed with SPSS version 29. Categorical variables were presented as frequencies and percentages, and continuous variables were summarized as mean ± SD or median (IQR), depending on normality. A paired *t*-test was used to compare pre- and post-test results. An unpaired *t*-test was used to compare pre- and post-test scores and change scores across baseline variables. Multiple linear regression analysis was conducted to identify variables influencing the change between pre- and post-test knowledge scores. Skill assessment scores before and after the intervention were analyzed using the Wilcoxon signed-rank test.

## Results

A total of 437 households were visited during the initial phase of the study, and 8.4% of households refused to participate. In the 400 participating households, 953 members were present at the time of the visit. One participant was selected from each household, yielding a final sample of 400 participants.

The mean age of participants was 46.11 years (SD 12.64), indicating variability in age within the sample. The mean age of males was 46.5 years, and the mean age of females was 45.9 years.

Table 1 shows that a higher proportion of females participated in the study. Only a small proportion of participants were illiterate; about three-fifths had attended school and one-third had completed a diploma, graduation, or higher. Half of the population had a monthly family income between INR 9221 and 15,368, with the remainder distributed on either side of this range.

**Table 1.** Frequency (%) of the baseline variables (*n* = 400).

Variable	Frequency (%)
Sex	
Male	123 (30.8%)
Female	277 (69.3%)
Education	
Illiterate	31 (7.8%)
Primary (1–4)	34 (8.5%)
Mid-primary (5–7)	91 (22.8%)
High school (8–10)	116 (29%)
Diploma	60 (15%)
Graduate & above	68 (17%)
Family Income/per month in Indian national rupees (INR) (Based on Modified Kuppuswamy classification with Consumer Price Index calculated as of November 2023).^21^	
3104 & below	21 (5.3%)
3105–9220	64 (16%)
9221–15,368	200 (50%)
15,369–23,052	59 (14.8%)
23,053–30,736	31 (7.8%)
30,737–61,473	16 (4%)
61,474 & above	9 (2.3%)

Most women in the study were engaged in household work, beedi rolling, or agricultural activities. Because the intervention was conducted during daytime hours, women were more likely than men to be present and enrolled.

Table 2 shows that the intervention has led to a substantial increase in participants’ first aid knowledge, evidenced by the statistically significant increase in post-intervention scores compared to pre-intervention. This could help the participant manage primary injuries and emergencies effectively. The significant improvement in knowledge scores after the intervention could also be due to potential encounters with episodes for which the training was given, which might reinforce their knowledge scores. It is also challenging to isolate the independent effect of providing kits and the education received on knowledge scores.

**Table 2.** Effect of health education on first aid knowledge (*n* = 400).

Knowledge score (80 points)	Mean ± SD	Mean difference (95% CI)	*t*-value^b^	*p*-value	Cohen’s *D*
Pre-intervention	26.85 ± 6.73	−35.85 (−36.73, −34.96)	−79.64	*p* < 0.001^a^	4.42^a^
Post-intervention (after 3 months)	62.69 ± 9.28				

a Statistically significant (*p* < 0.05).

b Paired *t*-test, Cohen’s *D* ≥ 0.8 indicates large effect size.

During the 3-month period between the intervention and the post-intervention assessment of knowledge and skills, trained household members encountered 126 episodes of minor wounds resulting in abrasions, 155 episodes of bleeding from wounds due to knife cuts and falls, 29 episodes of minor burns, 18 episodes of foreign bodies in the eye, and one episode of seizures.

Males and females had similar knowledge scores before the intervention. Three months after the intervention, knowledge scores had increased significantly in both groups; however, the increase was significantly greater among females than males (post-test *p* value = 0.044; change score *p* value = 0.008).

Participants aged ≤ 40 years had similar baseline knowledge scores to those aged > 40 years. After 3 months, scores increased significantly in both age groups, but the improvement was significantly greater among those aged ≤ 40 years (post-test *p* value = 0.002; change score *p* value = 0.02).

Participants with lower education levels (school education or uneducated) had significantly lower pre- and post-test knowledge scores than those with higher education (diploma or graduation), with a p value < 0.001. However, change in scores did not differ significantly between the two groups (*p* = 0.214), indicating a similar incremental increase in knowledge after the intervention across education levels.

A similar trend was observed for income: participants with lower income (<15,368 INR) had significantly lower pre- and post-test scores compared with those with higher income (≥15,368 INR), with p values of 0.008 and 0.016, respectively. However, the change in scores were not significantly different between income groups (*p* = 0.700). As with education, the impact of the intervention was similar across income levels, suggesting that it can be implemented in rural households regardless of income.

Participants who were unemployed or in elementary occupations had significantly lower pre- and post-test scores compared with those in other occupations (*p* = 0.002 and 0.018, respectively). However, change scores did not differ significantly between occupation groups (*p* = 0.910). As seen with education and income, the intervention had a similar impact across occupational categories (Table 3).

**Table 3.** Comparison of pre-intervention, post-intervention, and the difference in knowledge scores among baseline variables (*n* = 400).

Variable	Group	*N*	Pre-test (mean ± SD)	*p*-value	Post test (mean ± SD)	*p*-value	Change score (mean ± SD)	*p*-value
Sex	Male	123	27.13 ± 6.77	0.539	61.29 ± 10.60	0.044*	34.16 ± 9.69	0.008*
	Female	277	26.69 ± 6.62		63.31 ± 8.57		36.77 ± 8.69	
Age	≤ 40 years	130	27.27 ± 6.69	0.353	64.75 ± 8.78	0.002*	37.48 ± 8.20	0.020*
	>40 years	270	26.61 ± 6.65		61.70 ± 9.36		35.24 ± 9.40	
Education	Illiterates + school education	273	25.41 ± 6.08	*p* < 0.001*	60.85 ± 9.62	*p* < 0.001*	35.58 ± 9.29	0.214
	Diploma + graduation	127	29.87 ± 6.86		66.66 ± 7.04		36.80 ± 8.58	
Family income(INR)	<15,368	285	26.26 ± 6.62	0.008*	61.98 ± 9.59	0.016*	35.86 ± 9.07	0.700
	≥15,368	115	28.21 ± 6.57		64.45 ± 8.22		36.24 ± 9.13	
Occupation	Unemployed + elementary occupation	193	25.7 ± 5.98	0.002*	61.56 + 8.82	0.018*	36.02 + 8.50	0.910
	Others	207	27.83 + 7.10		63.75 + 9.59		35.92 + 9.60	

* *p* < 0.05, statistically significant, Unpaired t-test.

Table 4 presents the results of multiple linear regression predicting the difference between pre- and post-test scores based on sex, occupation, education, income, and age. The intercept represents the baseline mean difference in scores, with specific categories of each variable compared to a reference level. The adjusted *R*^2^ of 0.0219 indicates that the model explains approximately 2.19% of the variance in the change in scores.

**Table 4.** Multiple linear regression for predicting the variables for the difference in pre- and intervention knowledge scores.

			95% confidence interval			
Predictor	Estimate (β)	Standard Error (SE)	Lower	Upper	*t* value	*P* value
Intercept	35.0615	1.341	32.426	37.697	26.1533	<0.001
Sex: Female – Male^a^	2.8196	1.011	0.832	4.807	2.7893	**0.006**
Occupation group: Others – (unemployed + elementary occupation^a^)	0.2490	0.958	−1.634	2.132	0.2600	0.795
Education: diploma/graduation – illiterate/ school education^a^	0.8346	1.042	−1.213	2.882	0.8012	0.424
Family income: ≥15,368 – <15,368^a^	−0.0807	1.038	−2.122	1.961	−0.0777	0.938
Age: >40 years–≤40 years^a^	−2.0995	0.998	−4.062	−0.137	−2.1030	**0.036**

a Represents reference level, adjusted *R*^2^= 0.0219, *p*< 0.05 is statistically significant.

The coefficient for sex indicates that being female, compared to male, is associated with a 2.82-point increase in change scores (*p* = 0.006), suggesting that females showed significantly greater improvement than males. Coefficients for occupation, education, and income were not statistically significant, indicating no significant influence on knowledge scores after the intervention. Age was a significant predictor, with participants aged > 40 years showing smaller improvements compared with those aged ≤40 years.

Table 5 compares first aid skill assessment scores before and after the intervention. The Wilcoxon signed-rank test showed a statistically significant improvement in first aid skills after the intervention ( *p* value = 0.018). Participants were able to perform the skills acquired during training after 3 months, meeting one of the main objectives of the study. Provision of first aid kits to each household may have facilitated skill performance.

**Table 5.** Comparison of skill assessment scores on first aid between pre-intervention and post-intervention.

Skill assessment parameters (100 points)	Pre-intervention median scores	Post-intervention median scores	Pre-intervention total score median (Q1, Q3)	Post-intervention total score median (Q1, Q3)	Wilcoxon signed-rank test value	*p* value
Abrasion (18 points)	7.14 (39.7%)	15.12 (84%)	39.7 (37.8, 48.9)	87 (84, 88.9)	2.366	**0.018** *
Cut injury (18 points)	6.1 (33.8%)	16.02 (89%)				
Burns (18 points)	8.31 (46.2%)	15.66 (87%)				
Foreign body in the eye (8 points)	4.6 (57.5%)	7.2 (90%)				
ORS preparation (10 points)	3.75 (37.5%)	8.31 (83.1%)				
Laceration (18 points)	5.2 (28.9%)	16 (88.9%)				
Fall from height (10 points)	4.6 (46%)	8.79 (87.9%)				

* *p* < 0.05, statistically significant, Wilcoxon signed-rank test.

During the post-intervention assessment visit, participants were asked about their opinions regarding the usefulness of first aid training and provision of first aid boxes, using a structured questionnaire. Overall, 88.5% of participants reported that having a first aid box and training in every household is essential for preventing and managing common injuries and health-related events.

## Discussion

In this study, there was a statistically significant increase in first aid knowledge scores 3 months after training (*p* < 0.001). Similar improvements in knowledge after training have been reported in a study by Karaca and Kose, among 747 people without medical education.^22^ In contrast, a study by Andsoy et al. , among security personnel reported no significant improvement in knowledge scores, possibly because many participants already had prior first aid knowledge and training was delivered to large groups, unlike the individual household-based training in the present study.^23^ Significant improvements after first aid training were also observed in studies by Sonavane et al.,^24^ among mothers of children under 15 years in rural South India and by Omnia El Seifi et al.,^25^ among mothers with preschool children in Egypt, where mean knowledge scores increased from 10.21 ± 3.1 to 18.9 ± 2.6. Similar gains have been reported by Ygiyeva et al.,^26^ among workers without medical education, Miller and Newnam,^27^ among laypeople, Sarabi and Nosratabadi,^28^ among mothers, Vahdaninya et al.,^29^ and Shah et al.,^30^ among tribal women in India. A systematic review by Van de Velde et al. , concluded that first aid training improves knowledge and skills and may overcome barriers to emergency helping behavior.^31^ Another systematic review by Strømme et al. , also reported improvements in first aid knowledge and skills after training, at least in the short term.^32^ Taken together with the present findings, these studies confirm that well-planned and implemented first aid training, even among those without a medical background, can significantly improve knowledge and confidence in handling medical emergencies.

In the present study, statistically significant improvements in knowledge scores were observed across age, sex, education, family income, and occupation. The increase was greater among participants younger than 40 years and among females. This could be because women in these households are more often exposed to situations requiring first aid and may therefore be more receptive and attentive during training. Regression analysis also showed that sex and age significantly predicted changes in scores, with females and younger participants demonstrating greater improvements. Occupation, education, and income did not significantly influence the change in scores, suggesting that first aid health education is effective across these strata. Similar findings were reported in a study done by Serinken et al.^33^ In contrast, Selda Mert-Boga et al.,^34^ found greater improvement among participants with higher education, a pattern not seen in the present study.

In this study, post-intervention skill assessment scores were higher for all parameters and were statistically significant, consistent with findings from Joys et al.,^35^ A systematic review by Aaron M Orkin et al. , on emergency care delivered by lay responders in underserved populations concluded that first aid training for laypeople has greater impact when tailored to emergencies they are likely to encounter in their daily lives and communities.^36^ The present health education model can be implemented at the primary health care level, as training in this study was provided by qualified nurses who are readily available at primary health centers, with appropriate local adaptations. If implemented on a large scale, such training can empower communities to provide emergency care during disasters such as earthquakes, landslides, floods, epidemics, or pandemics, thereby saving lives until hospital care or emergency medical teams are available.

One important challenge in the consistent application of first aid knowledge and skills is limited practice and poor skill retention. Current literature suggests that first aid skills begin to deteriorate within 2–6 months after training.^37–40^ For example a study by Anderson et al. , showed that many skills decline rapidly over 3 months, highlighting the need for regular refresher sessions.^41^ The consensus across multiple studies is that regular refresher training is essential to counteract knowledge and skill decay and to ensure that trained individuals remain capable of providing effective first aid.^26,37,39,40^ Future research could also focus on other domains, such as cardiopulmonary resuscitation and early detection of stroke.

One limitation of this study is the absence of a control group that did not receive the intervention. Although a before-after design provides information on baseline knowledge and changes following training, confounding variables unrelated to the intervention may influence the outcomes. A more comprehensive assessment of impact would be possible with one control group receiving no education or first aid kit and another receiving education only without the kit. Another limitation is that the study relied on checklists and simulations to estimate the intervention’s impact, which may overestimate performance during real emergencies.

## Conclusion

The intervention significantly improved participants’ knowledge of how to handle emergencies, as demonstrated by the marked increase in post-intervention test scores compared with pre-test scores. Initially, participants younger than 40 years, females, and those with higher education and income had higher pre-test scores. However, after the intervention, knowledge improved across all groups. This suggests that a well-designed health education module, combined with provision of first aid kits, effectively enhances knowledge and skills in managing emergencies regardless of participants education, occupation, or income. These findings emphasize the importance of targeted first aid training to strengthen emergency preparedness across populations.

## Supplemental Material

sj-docx-1-phj-10.1177_22799036261427950 – Supplemental material for Evaluation of the effectiveness of health education on first aid with the provision of first aid kits among rural households of India: A quasi-experimental study Supplemental material, sj-docx-1-phj-10.1177_22799036261427950 for Evaluation of the effectiveness of health education on first aid with the provision of first aid kits among rural households of India: A quasi-experimental study by Amit Kumar Rao, Rahul Hegde, Nanjesh Kumar Siddappa, Abiga George, Sanjay Kini Bailur and Harshitha Hanglur Narasimha in Journal of Public Health Research

sj-pdf-2-phj-10.1177_22799036261427950 – Supplemental material for Evaluation of the effectiveness of health education on first aid with the provision of first aid kits among rural households of India: A quasi-experimental study Supplemental material, sj-pdf-2-phj-10.1177_22799036261427950 for Evaluation of the effectiveness of health education on first aid with the provision of first aid kits among rural households of India: A quasi-experimental study by Amit Kumar Rao, Rahul Hegde, Nanjesh Kumar Siddappa, Abiga George, Sanjay Kini Bailur and Harshitha Hanglur Narasimha in Journal of Public Health Research

## Appendix

**Appendix 1.** Contents of Identical First Aid Kits provided to each household.

Sl. No	Particulars	Quantity
1	Cotton balls	1 packet
2	Roller bandage (10 cm × 3 m)	1
3	Roller bandage (5 cm × 3 m)	1
4	Gauze pad (10 cm × 10 cm)	1 packet
5	Gauze pad (5 cm × 5 cm)	1 packet
6	Microporous tape	1
7	Burn dressing	1
8	Antiseptic cream	1
9	Antiseptic solution (50 ml)	1
10	Sterile dressing (17 cm × 17 cm)	1
11	Plasters	10
12	Scissor	1
13	Eye pad	2
14	Oral rehydration salts	2 sachets
15	First aid ready reckoner (English, Kannada and Malayalam)	1
16	Emergency telephone number of the nearby hospital/health center	
