# Supplementary material for: Evaluation of the effectiveness of health education on first aid with the provision of first aid kits among rural households of India: A quasi-experimental study
Source: J Public Health Res. 2026 Mar 23;15(1):22799036261427950. doi: 10.1177/22799036261427950 (PMC13009947; doi:10.1177/22799036261427950)
Supplement: sj-docx-1-phj-10.1177_22799036261427950 – Supplemental material for Evaluation of the effectiveness of health education on first aid with the provision of first aid kits among rural households of India: A quasi-experimental study [file sj-docx-1-phj-10.1177_22799036261427950.docx]

**INFORMED CONSENT**

**Study title:** Study to evaluate the effectiveness of Health education on First aid with provision of First aid kit among rural households of Coastal Karnataka.

I, ____________________________________________ (Participant’s name), have understood the information given by the researcher on the nature and the objective of the study titled above. I have been informed what I have to do as a part of the study. I have had the time and opportunity to enquire about the study and I am fully satisfied with the explanations given.

I understand that I am at liberty to withdraw from this study at any time without justifying my decision to withdraw.

I know that the results from this study may be forwarded to the appropriate authorities, presented in scientific meetings, and published.

By signing this consent form, I have not given up any legal rights that I am otherwise entitled to as a subject in this study.

I acknowledge that I will get a copy of this consent form which is signed and dated.

____________________________

Signature of the study participant.

**ಮಾಹಿತಿ ಒಪ್ಪಿಗೆ ಪತ್ರ**

ಅಧ್ಯಯನದ ಶೀರ್ಷಿಕೆ: ಕರಾವಳಿ ಕರ್ನಾಟಕದ ಗ್ರಾಮೀಣ ಮನೆಗಳಲ್ಲಿ ಪ್ರಥಮ ಚಿಕಿತ್ಸಾ ಕಿಟ್ ಒದಗಿಸುವುದರೊಂದಿಗೆ ಪ್ರಥಮ ಚಿಕಿತ್ಸೆಯಲ್ಲಿ ಆರೋಗ್ಯ ಶಿಕ್ಷಣದ ಪರಿಣಾಮಕಾರಿತ್ವವನ್ನು ಮೌಲ್ಯಮಾಪನ ಮಾಡಲು ಅಧ್ಯಯನ.

ನಾನು, ________________________________________________________________________ (ಭಾಗವಹಿಸುವವರ ಹೆಸರು), ಮೇಲಿನಂತೆ ಶೀರ್ಷಿಕೆಯ ಅಧ್ಯಯನದ ಸ್ವರೂಪ ಮತ್ತು ಉದ್ದೇಶದ ಕುರಿತು ಸಂಶೋಧಕರು ನೀಡಿದ ಮಾಹಿತಿಯನ್ನು ಅರ್ಥಮಾಡಿಕೊಂಡಿದ್ದೇನೆ. ಅಧ್ಯಯನದ ಭಾಗವಾಗಿ ನಾನು ಏನು ಮಾಡಬೇಕೆಂದು ನನಗೆ ತಿಳಿಸಲಾಗಿದೆ. ಅಧ್ಯಯನದ ಬಗ್ಗೆ ವಿಚಾರಿಸಲು ನನಗೆ ಸಮಯ ಮತ್ತು ಅವಕಾಶವಿದೆ ಮತ್ತು ನೀಡಿರುವ ವಿವರಣೆಗಳಿಂದ ನಾನು ಸಂಪೂರ್ಣವಾಗಿ ತೃಪ್ತನಾಗಿದ್ದೇನೆ.

ಹಿಂತೆಗೆದುಕೊಳ್ಳುವ ನನ್ನ ನಿರ್ಧಾರವನ್ನು ಸಮರ್ಥಿಸದೆ ಯಾವುದೇ ಸಮಯದಲ್ಲಿ ಈ ಅಧ್ಯಯನದಿಂದ ಹಿಂದೆ ಸರಿಯಲು ನನಗೆ ಸ್ವಾತಂತ್ರ್ಯವಿದೆ ಎಂದು ನಾನು ಅರ್ಥಮಾಡಿಕೊಂಡಿದ್ದೇನೆ.

ಈ ಅಧ್ಯಯನದ ಫಲಿತಾಂಶಗಳನ್ನು ಸೂಕ್ತ ಅಧಿಕಾರಿಗಳಿಗೆ ರವಾನಿಸಬಹುದು, ವೈಜ್ಞಾನಿಕ ಸಭೆಗಳಲ್ಲಿ ಪ್ರಸ್ತುತಪಡಿಸಬಹುದು ಮತ್ತು ಪ್ರಕಟಿಸಬಹುದು ಎಂದು ನನಗೆ ತಿಳಿದಿದೆ.

ಈ ಸಮ್ಮತಿಯ ನಮೂನೆಗೆ ಸಹಿ ಹಾಕುವ ಮೂಲಕ, ಈ ಅಧ್ಯಯನದಲ್ಲಿ ವಿಷಯವಾಗಿ ನಾನು ಅರ್ಹರಾಗಿರುವ ಯಾವುದೇ ಕಾನೂನು ಹಕ್ಕುಗಳನ್ನು ನಾನು ಬಿಟ್ಟುಕೊಟ್ಟಿಲ್ಲ.

ನಾನು ಈ ಸಮ್ಮತಿಯ ನಮೂನೆಯ ನಕಲನ್ನು ಸಹಿ ಮತ್ತು ದಿನಾಂಕವನ್ನು ಪಡೆಯುತ್ತೇನೆ ಎಂದು ಒಪ್ಪಿಕೊಳ್ಳುತ್ತೇನೆ.

ಅಧ್ಯಯನದಲ್ಲಿ ಭಾಗವಹಿಸುವವರ ಸಹಿ.
