# Supplementary material for: Evaluation of the effectiveness of health education on first aid with the provision of first aid kits among rural households of India: A quasi-experimental study
Source: J Public Health Res. 2026 Mar 23;15(1):22799036261427950. doi: 10.1177/22799036261427950 (PMC13009947; doi:10.1177/22799036261427950)
Supplement: sj-pdf-2-phj-10.1177_22799036261427950 – Supplemental material for Evaluation of the effectiveness of health education on first aid with the provision of first aid kits among rural households of India: A quasi-experimental study [file sj-pdf-2-phj-10.1177_22799036261427950.pdf]

icmr
INDIAN COUNCIL OF
MEDICAL RESEARCH
Serving the nation since 1911

भारतीय आयुर्विज्ञान अनु[illegible]
स्वास्थ्य अनुसंधान विभाग, स्वास्थ्य [illegible]
कल्याण मंत्रालय, भारत [illegible]

Indian Council of Medical Research
Department of Health Research, Ministry of Health
and Family Welfare, Government of India

File No5/4-5/3/8/Trauma/2022/NCD-I

Date: 16.03.2023

Subject: - Payment of full & final installment of the 1st year grant-in-aid for the project entitled "Study to evaluate the effectiveness of Health education on First aid with provision of First aid kit among rural households of Coastal Karnataka" under **Dr. Amit Kumar Rao, K.S. Hegde Medical Academy, Deralakatte, Mangalore, Karnataka.**

MAMORANDUM

The Director General of the ICMR sanctions the grant of **Rs. 16,43,120/-** (Rupees sixteen lakh forty three thousand one hundred twenty only) as the full& final installment of the 1st year grant for the period from **24.03.2023 to 23.03.2024** for incurring expenditure in connection with the above mentioned project. The amount of **Rs. 16,43,120/-** may be debited to the provision made of **Rs. 16,43,120/-** on the above mentioned research project for the year 2022-2023.

A formal bill for **Rs. 16,43,120/-** is sent herewith for payment release by NEFT/RTGS in favour of the NITTE DEEMED TO BE UNIVERSITY Deralakatte, Mangalore -575018.

This issued with the concurrence of the Finance Division, vide RFC No. (P-69) NCD/Adhoc/230/2022-23 Dated 14.03.2023

(Harjeet Kaur Bajaj)
Administrative Officer
For Director General

Copy to:

1. The Dean, K.S. Hegde Medical Academy, Deralakatte, Mangalore, Karnataka
2. Dr. Amit Kumar Rao, Assistant Professor, Department of Community Medicine, K.S. Hegde Medical Academy, Deralakatte, Mangalore, Karnataka. It is requested that an audited statement of account together with utilization certificate of the grant received and utilized in last year grant may kindly be sent to this office.
3. Accounts -V
4. IRIS Cell No. 2021-13779
5. Mr. Hemant Kumar, Sr. Technical Officer

For Director General

INDIAN COUNCIL OF MEDICAL RESEARCH
Serving the nation since 1911

भारतीय आयुर्विज्ञान अनुसंधान परिषद
स्वास्थ्य अनुसंधान विभाग, स्वास्थ्य और परिवार कल्याण मंत्रालय, भारत सरकार

Indian Council of Medical Research
Department of Health Research, Ministry of Health and Family Welfare, Government of India

File No.5/4-5/3/8/Trauma/2022/NCD-I

Date: 16/03/2023

To,

The Dean,
KS, Hegde Medical Academy,
Deralakatte Post, Nithyanandanagara
Mangalore-575018
Karnataka

Subject: -Sanction of budget allotment for the Ad-hoc project entitled "Study to evaluate the effectiveness of Health education on First aid with provision of First aid kit among rural households of Coastal Karnataka" under **Dr. Amit Kumar Rao, K.S. Hegde Medical Academy, Deralakatte, Mangalore, Karnataka.**

Sir/Madam,

The Director General, ICMR has been sanctioned the above-mentioned research proposal for the period of **2 years** with budget **of Rs. 16,43,120/-** (Rupees sixteen lakh forty three thousand one hundred twenty only). This proposal has been sanctioned initially for **one year** from **24.03.2023 to 23.03.2024** and it may be extended on the yearly basis after review of the work done during the period. The grant-in-aid will be given subject to the following condition.

1. The payment of the grant will be made in lump-sum to the Head of the Institute. The first installment of the grant will be paid generally as soon as report regarding appointment of the staff is received by the Council. The Staff appointed on the project should be paid as indicated in the budget statement.
2. The staff on the project will be recruited as per the rules and procedure of the host institute and second part of the undertaking be obtained from the employees of the project. The staff grant will not be released unless the required undertaking [part-II] from Head of the Institute is received in this office.
3. The demand for payment of the subsequent installment of the grant should be placed with the Council in the prescribed Performa.
4. Five copies of the annual progress report should be submitted to the ICMR every year after completion of ten months of the project giving complete actual details of the research work done. Failure to submit the report in time may lead to termination of project.
5. Subject to the condition that the grant will be utilize after following the provisions laid down in the GFRs-2017 & TA Rules.
6. Please keeps the fund in the separate saving Bank Account opened for ICMR funded Research projects so that interest earned thereon is credited into the accounts.
7. In the case of JRF – after completion of two years, an external assessment by the Institution, where the student is enrolled for Ph.D, is mandatory for upgradation from JRF to SRF. The fellow may be upgraded as SRF after successful assessment.
8. The receipt of this letter may please be acknowledged.

Yours faithfully,

for [signature]
(Harjeet Kaur Bajaj)
Administrative Officer
For Director General

Copy together with a copy of the budget statement forwarded to information to:-

1. **Dr. Amit Kumar Rao,** Assistant Professor, Department of Community Medicine, K.S. Hegde Medical Academy, Deralakatte, Mangalore, Karnataka.
2. Accounts- V. for information.
3. IRIS Cell No.2021-113779
4. Mr. Hemant Kumar, Sr. Technical Officer

For Director General

Project entitled "Study to evaluate the effectiveness of Health education on First aid with provision of First aid kit among rural households of Coastal Karnataka" under **Dr. Amit Kumar Rao, K.S. Hegde Medical Academy, Deralakatte, Mangalore, Karnataka.**

Budget for 1st year
24.03.2023 to 23.03.2024

Particulars	Budget
	1st Year
A. Manpower/ Staff	
**Social Worker (Two)**<br>Rs. 32,000/- x 2<br><br>10% annual increment @ Rs. 1390/- added in further each year as per ICMR guidlines	768000
**Junior Nurse (Two)**<br>Rs. 18,000/- x 2<br><br>10% annual increment @ Rs. 800/- added in further each year as per ICMR guidlines	432000
**Data Entry Operator/ Data Manager (One)**<br>Rs. 17,000/-<br><br>**(1st Year 2 Months & 2nd Year 2 Months)**<br><br>10% annual increment @ Rs. 760/- added in further each year as per ICMR guidlines	34000
**Sub Total –A**	**1234000**
**B. Contingencies**<br>Stationeries, training and monthly meeting	70000
**C. Overhead charges (3%) (A+B)**	**39120**
**D. Equipments**<br>i) First aid kits: 400 kits (Rs. 500 each)<br>ii) Electronic tablets: 2 tablets (Rs. 12,500 each)	225000
**E. Travel**	75000
**Grand Total (A+B+C+D+E)**	**1643120**

for [signature]
Sr. Administrative Officer
For Director General

NITTE (DEEMED TO BE UNIVERSITY)
6TH FLOOR, MEDICAL SCIENCES COMPLEX, DERALAKATTE
MANGALORE
575018

Website : www.nitte.edu.in
Email : info@nitte.edu.in

Receipt No.: 225102884 PAN No.: 1 Date: 31-03-2023

Name: Indian Council Of Medical Research / Icmr

Remarks:

2255259	ICMR-Dr Amit Kumar Rao, KSHEMA	1643120.00

Total: 1643120.00

Amount in words: Rupees Sixteen Lakh Forty Three Thousand One Hundred Twenty Only.

DD/Cheque Details: NEFT RBI0802363437061 20/03/2023 1643120.00

Suma

Cashier

Note: Cheque/DD subject to realisation.

ac70a87876e83ca3df7a9092e3c1cc8739ad9eab750a30a7a86bba7c6d9c6ddaa8ac35ae4da6eba0b19b
